# Comprehensive Safety Assessment of *Lentilactobacillus buchneri* KU200793 as a Potential Probiotic

**DOI:** 10.3390/microorganisms13092067

**Published:** 2025-09-05

**Authors:** Suin Kim, Huijin Jeong, Na-Kyoung Lee, Dae-Kyung Kang, Hyun-Dong Paik, Young-Seo Park, Jong Hun Lee

**Affiliations:** 1 Department of Food Science and Biotechnology, Gachon University, Seongnam 13120, Republic of Korea; sue030465@gachon.ac.kr (S.K.); huijin0218@gmail.com (H.J.); 2 Department of Food Science and Biotechnology of Animal Resource, Konkuk University, Seoul 05029, Republic of Korea; nakyoung_lee@nate.com (N.-K.L.); hdpaik@konkuk.ac.kr (H.-D.P.); 3 Department of Animal Biotechnology, Dankook University, Cheonan 31116, Republic of Korea; dkkang@dankook.ac.kr

**Keywords:** genotypic analysis, *Lentilactobacillus buchneri*, neuroprotective effects, phenotypic analysis, probiotics, safety evaluation

## Abstract

The safety profile of *Lentilactobacillus buchneri* KU200793, which has neuroprotective effects, was comprehensively evaluated through both phenotypic and genotypic analyses. Phenotypically, the strain exhibited no β-hemolysis, mucin degradation, indole production, gelatin liquefaction, urease activity, or β-glucuronidase activity. Additionally, it did not produce D-lactate, and only trace amounts of spermidine were detected among the biogenic amines. Furthermore, *L. buchneri* KU200793 did not exhibit bile salt deconjugation, further supporting its safety profile. However, its tetracycline resistance exceeded the threshold set by the European Food Safety Authority. Genotypic analysis using the HGTree program identified *tetA*(*58*) and *nalD* genes with sequence similarities of 33.64% and 30.17%, respectively, indicating a low level of homology. These findings suggest that tetracycline resistance in *L. buchneri* KU200793 is unlikely to have been acquired through horizontal gene transfer, thereby minimizing the risk of resistance gene dissemination. This study underscores the importance of comprehensive safety assessments to evaluate the suitability of *L. buchneri* KU200793 for probiotic applications.

## 1. Introduction

Lactic acid bacteria (LAB) have long been utilized as probiotics in dietary supplements and food products [1]. LAB serve not only as natural preservatives by enhancing the flavor and acidity of food but also offer various health benefits, including improving mucosal integrity, exhibiting anti-pathogenic properties, aiding in the management of lactose intolerance and food allergies, preventing diarrhea, and alleviating inflammation [2]. The Food and Agriculture Organization (FAO) and the World Health Organization (WHO) defined probiotics as “live microorganisms that, when administered in adequate amounts, confer a health benefit on the host” [3]. Traditionally, probiotics have been studied for their role in immune modulation and gut health. Recently, growing research into the causal links between microbiota and various diseases has increased interest in the therapeutic potential of probiotics for disease management [4,5].

Despite their potential health benefits, the expanding diversity of LAB and their increasing use as probiotics, particularly strains belonging to the genus *Lactobacillus*, have raised concerns regarding their safety [6,7]. A report by FAO and WHO highlights four potential adverse effects of probiotics: invasive infections, harmful metabolic processes, excessive immune activation in vulnerable individuals, and horizontal gene transfer (HGT) [8]. Furthermore, certain LAB are opportunistic pathogens linked to bacteremia, a rare but increasingly observed condition in patients with immune disorders or underlying health conditions. These concerns underscore the need for comprehensive safety assessments to ensure the safe use of LAB in food and dietary supplements [9].

*Lentilactobacillus buchneri* is an obligate heterofermentative, facultative anaerobic bacterium naturally found in diverse ecological environments and is closely associated with food production and fermentation processes [10]. This species is well-known for its beneficial effects, including the conversion of lactic acid to acetic acid, which inhibits the growth of certain yeasts and molds [11]. *L. buchneri* KU200793 exhibits various potential probiotic properties, including high resistance to artificial gastric juice and bile salts, cholesterol-lowering effects, and antimicrobial activity. Additionally, this strain demonstrates beneficial β-galactosidase activity and the absence of β-glucuronidase, indicating its safety. It also demonstrates remarkable antioxidant activity under heat-killed conditions. Furthermore, *L. buchneri* KU200793 has been shown to protect SH-SY5Y neuronal cells from MPP^+^-induced neurotoxicity by significantly enhancing cell viability and upregulating brain-derived neurotrophic factor (BDNF) mRNA expression. It also reduces the Bax/Bcl-2 ratio, thereby exerting an anti-apoptotic effect. These findings suggest that *L. buchneri* KU200793 possesses neuroprotective potential through the gut–brain axis and may serve as a promising probiotic candidate for the prevention of neurodegenerative diseases [12].

Although this strain was originally isolated from kimchi, a traditional fermented food, *L. buchneri* KU200793 has not been intentionally applied or evaluated for use in commercial food products or dietary supplements. Considering its potential application as a dietary supplement for humans, a comprehensive safety assessment is essential [13]. Accordingly, this study aimed to evaluate the safety of *L. buchneri* KU200793 based on the guidelines provided by the FAO/WHO (2002) and European Food Safety Authority (EFSA) [14].

## 2. Materials and Methods

### 2.1. Bacterial Strains and Culture Conditions

*L. buchneri* KU200793 (KCCM 12593P) was obtained from Konkuk University (Seoul, Republic of Korea) and grown anaerobically in de Man, Rogosa, and Sharpe broth (MRS; BD, Franklin Lakes, NJ, USA) at 37 °C for 24 h. The MRS broth contained (per liter): 10 g of protease peptone No. 3, 10 g of beef extract, 5 g of yeast extract, 20 g of dextrose, 1 g of polysorbate 80, 2 g of ammonium citrate, 5 g of sodium acetate, 0.1 g of magnesium sulfate, 0.05 g of manganese sulfate, and 2 g of dipotassium phosphate.

*Klebsiella pneumoniae* subsp. *pneumoniae* KCCM 60022, *Proteus vulgaris* KCCM 40221, *Escherichia coli* KCCM 11234, *Streptococcus pneumoniae* KCCM 41570, *S. pyogenes* KCCM 40411, *S. agalactiae* KCCM 40417, and *Brevibacillus* (*Br.*) *parabrevis* KCCM 41421 were purchased from the Korean Culture Center of Microorganisms (Seoul, Republic of Korea). *Lactiplantibacillus plantarum* KU15122 was obtained from Konkuk University (Seoul, Republic of Korea). *Bacillus cereus* KCCM 11204, *K. pneumoniae* subsp. pneumoniae KCCM 60022, *P. vulgaris* KCCM 40221, *E. coli* KCCM 11234, and *Br. parabrevis* KCCM 41421 were cultured in nutrient broth (BD) (3 g of beef extract and 5 g of peptone, per liter) at 30 °C for 24 h. *Lp. plantarum* KU15122 was cultured in MRS medium at 37 °C for 24 h.

### 2.2. Cell Culture Conditions

The Caco-2 cell line (ATCC^®^ HTB-37™) was obtained from the American Type Culture Collection (ATCC, Manassas, VA, USA). Caco-2 cells are widely used as an in vitro model of intestinal epithelial cells in probiotic safety assessments, as their viability reflects the absence of cytotoxic effects. The cells were cultured in an incubator (Thermo Fisher Scientific, Waltham, MA, USA) maintained at 37 °C with 5% CO_2_. Dulbecco’s modified Eagle’s medium (DMEM; Gibco, Grand Island, NY, USA) supplemented with 1% (*v*/*v*) penicillin–streptomycin (Gibco), and 10% (*v*/*v*) fetal bovine serum (FBS; Corning Inc., Corning, NY, USA) was used as the culture medium.

### 2.3. Assessment of Hemolytic Activity

Hemolytic activity was evaluated by streaking bacteria on blood agar base No. 2 (Sigma-Aldrich, St. Louis, MO, USA) supplemented with 5% sheep blood (KisanBio, Seoul, Republic of Korea). The plates were inoculated with *L. buchneri* KU200793, *S. pneumoniae* KCCM 41570, *S. pyogenes* KCCM 40411 and *S. agalactiae* KCCM 40417 and then incubated under anaerobic conditions at 37 °C for 48 h. Strains that formed a green area around the colony were classified as α-hemolytic, those that formed a clear zone were classified as β-hemolytic, and those with no observable color change around the colony were classified as γ-hemolytic.

### 2.4. Evaluation of Bile Salt Deconjugation

Bile salt deconjugation was assessed using MRS containing 0.5% taurodeoxycholic acid (bile acid; Sigma-Aldrich). Plates inoculated with *L. buchneri* KU200793 and *B. cereus* KCCM 11204 were anaerobically cultured at 37 °C for 48 h. Strains with sedimentation around the bacterial colonies were classified as exhibiting bile salt hydrolase (BSH) activity.

### 2.5. Cytotoxicity Assay Using Caco-2 Cells

Caco-2 cells were seeded at a density of 5 × 104 cells/well in a 96-well plate in DMEM supplemented with 10% FBS and 1% penicillin–streptomycin. The cells were incubated at 37 °C in a 5% CO_2_ incubator for 20 h. *L. buchneri* KU200793 was cultured in MRS broth at 37 °C for 18 h, while *K. pneumoniae* subsp. *pneumoniae* KCCM 60022, used as a positive control, was grown in nutrient broth under the same conditions. Overnight cultures were inoculated into 10 mL of MRS medium at 37 °C for 7 h. Cells were harvested by centrifugation at 16,000× *g* for 1 min. The harvested cells were washed thrice with Dulbecco’s phosphate-buffered saline (DPBS; Welgene, Gyeongsan, Republic of Korea) and resuspended in 1 mL of DMEM containing 10% (*v*/*v*) FBS and 1% penicillin–streptomycin [15]. This suspension was added to Caco-2 cells at a multiplicity of infection (MOI; the ratio of LAB cells to Caco-2 cells) of 31.3, 62.5, and 125, and the cultures were incubated at 37 °C in a 5% CO_2_ incubator for 24 h. After 24 h, plates were washed twice with DPBS. Subsequently, 200 μL of DMEM containing 10% FBS and 1% penicillin–streptomycin was added to each well, followed by 20 μL of EZ-Cytox solution (DoGenBio Co., Ltd., Seoul, Republic of Korea). The mixture was incubated at 37 °C in a 5% CO_2_ atmosphere for 30 min. The EZ-Cytox assay utilizes water-soluble tetrazolium-8, which is reduced by intracellular dehydrogenases in viable cells to produce a water-soluble formazan dye. The amount of formazan formed is proportional to the metabolic activity of viable cells and thus serves as an indirect indicator of cytotoxicity [16]. Absorbance at 450 nm, reflecting cell viability through the amount of formazan produced, was measured using Take3 Micro-Volume Plates (Epoch Microplate Reader; BioTek Instruments, Inc., Winooski, VT, USA).

### 2.6. Determination of Mucin Degradation

*L. buchneri* KU200793 was inoculated into four types of media: a basal medium without any carbon source, a basal medium with 0.3% (*w*/*v*) mucin as the sole carbon source, a basal medium supplemented with 1% (*w*/*v*) glucose, and a basal medium containing both 0.3% (*w*/*v*) mucin and 1% (*w*/*v*) glucose. Bacterial growth was assessed after 8 and 24 h of incubation by measuring the optical density (OD) at 600 nm using a spectrophotometer (BioSpec-mini, Shimadzu Co., Kyoto, Japan) and by monitoring pH changes. Growth in mucin-containing media was considered to indicate the strain’s ability to utilize mucin as a carbon source, thereby reflecting potential mucin-degrading activity.

### 2.7. Quantification of D-Lactic Acid Production

The LAB culture was centrifuged at 16,000× *g* for 10 min, and the supernatant was collected. The obtained supernatant was analyzed using an Enzytec™ Liquid D-Lactic acid assay kit (R-Biopharm, Darmstadt, Germany) following the manufacturer’s instructions. The D-lactic acid concentration was determined by measuring absorbance at 340 nm using a BioSpec-mini spectrophotometer (Shimadzu Co.). This assay specifically detects D-lactic acid without cross-reactivity to L-lactic acid.

### 2.8. Analysis of Biogenic Amine Production

*L. buchneri* KU200793 was cultured in MRS medium supplemented with 400 ppm each of amino acid precursors, including ornithine, histidine, tryptophan, tyrosine, lysine, arginine, and phenylalanine (Sigma-Aldrich) at 37 °C for 20 h. One milliliter of the culture supernatant, 500 μL of saturated sodium carbonate solution, and 800 μL of 1% (*w*/*v*) dansyl chloride acetone solution were mixed and reacted at 45 °C for 1 h. Subsequently, 500 μL of 10% (*w*/*v*) proline and 5 mL of diethyl ether were added to the derivatized mixture, which was shaken for 10 min. The supernatant was collected and evaporated under nitrogen gas, then dissolved in 1 mL of acetonitrile and filtered through a 0.22 μm polyvinylidene difluoride filter. The derivatized biogenic amine (BA) mixture was quantitatively analyzed by high-performance liquid chromatography using a DIONEX UltiMate 3000 system (Thermo Fisher Scientific). The analysis was performed using a 250 × 4.6 mm, 5 μm, Agilent 5 TC-C18 column (Agilent Technologies, Amstelveen, The Netherlands) and a UV detector set at 254 nm, at a column oven temperature of 40 °C. Twenty microliters of the sample was injected into the column at 1 mL/min in a mobile phase consisting of 55:45 (acetonitrile–water) after equilibration for 30 min. The standard BAs evaluated in this study were agmatine, β-phenylethylamine, histamine, putrescine, serotonin, spermidine, tryptamine, and tyramine. To complement the phenotypic data, genes related to the polyamine transport system (*potA–E*) were identified by keyword searching annotated coding sequences in the *L. buchneri* KU200793 genome.

### 2.9. Antibiotic Susceptibility Test

The antibiotic susceptibility of *L. buchneri* KU200793 was assessed using E-test strips (ETEST, bioMérieux, Marcy-l’Étoile, France). The minimum inhibitory concentration (MIC) of each antibiotic was determined based on the cut-off values established by the EFSA for probiotics [14]. A suspension of bacterial colonies was prepared in 0.88% (*w*/*v*) NaCl solution and adjusted to match the 0.5 McFarland standard. This suspension was uniformly spread onto the surface of agar plates using sterile cotton swabs. E-test strips for antibiotics were placed on the agar surface using sterile forceps, and the plates were incubated at 37 °C for 24 h before evaluating the MIC.

### 2.10. Genomic Analysis of Antibiotic Resistance and Virulence Factors

The whole-genome sequence of *L. buchneri* KU200793 (NCBI Bioproject accession number: PRJNA1237560) was provided by Dankook University and was used to identify antibiotic resistance. The analysis was performed using Resistance Gene Identifier (RGI) software version 6.0.3 (https://card.mcmaster.ca/analyze/rgi, accessed on 18 February 2025) based on the Comprehensive Antibiotic Resistance Database version 3.2.8 and ResFinder version 4.4.1 (http://genepi.food.dtu.dk/resfinder, accessed on 4 March 2025) based on the ResFinder database. These tools were used to examine the presence of antibiotic resistance genes (ARGs) and assess the resistance levels associated with mutations. The criteria for RGI analysis included the following: Selection criteria: Perfect, Strict, and Loose hits; Nudge ≥ 95% identity for Loose hits to Strict (excluding nudge and low-confidence hits); and Sequence Quality: high quality/coverage. Loose hits were considered indicative of ARGs if they exhibited ≥70% sequence identity. For ResFinder analysis, the threshold criteria were set at 90% sequence similarity and a minimum length of 60%. Additionally, HGT potential was analyzed using the HGTree2 program, whereas the presence of ARGs within the plasmids and their potential for horizontal transfer were assessed using PlasmidFinder 2.0. PHASTEST was used to identify prophage regions within the genome to assess whether transduction was involved in the horizontal transfer of resistance genes. The involvement of transposons in HGT was assessed using MobileElementFinder.

### 2.11. Gelatin Liquefaction Assay

The gelatin liquefaction assay was conducted in MRS medium supplemented with 12% (*w*/*v*) gelatin. A single colony of *L. buchneri* KU200793 was introduced into the medium and incubated at 37 °C for 72 h. *Br. parabrevis* KCCM 41421 was used as a positive control and was incubated under identical conditions. After incubation, the test tubes were placed in an ice bath for 30 min. Strains capable of liquefying gelatin were considered positive for gelatinase activity.

### 2.12. Urease Activity Assay 

The urease activity of bacterial cells was assessed by streaking them onto Christensen’s urea agar (0.1 g of peptone, 0.1 g of dextrose, 0.5 g of sodium chloride, 0.2 g of monopotassium phosphate, 2 g of urea, 0.012 g of phenol red) slants. *L. buchneri* KU200793 and *P. vulgaris* KCCM 40221, which was used as a positive control, were incubated at 37 °C for six days. Urease activity was determined by observing the changes in pH, as indicated by a color change in the slants from orange to pink. Pink coloration indicated ammonia production, confirming a positive urease reaction.

### 2.13. Indole Production Assay 

Indole production was assessed using tryptophan as a substrate. *L. buchneri* KU200793 and *E. coli* KCCM 11234, used as a positive control, were cultured in tryptophan broth (10.0 g of casein enzyme hydrolysate, 5.0 g of NaCl, 1.0 g of DL-tryptophan, per liter) at 37 °C for 24 h. After incubation, five drops of Kovac reagent (Sigma-Aldrich) were added to the medium. A red ring on the surface of the medium indicated a positive result for indole production.

### 2.14. Statistical Analysis

All experiments were conducted in triplicate, with the results expressed as the mean ± standard deviation (SD). Statistical analyses were performed using SPSS 28 (SPSS Inc., Chicago, IL, USA) software. One-way analysis of variance (ANOVA) was used to evaluate the statistical significance of the results, and Duncan’s multiple range test was used to identify significant differences between groups. A *p*-value of <0.05 was considered statistically significant.

## 3. Results

### 3.1. Hemolytic Profile of L. buchneri KU200793

**Figure 1:**
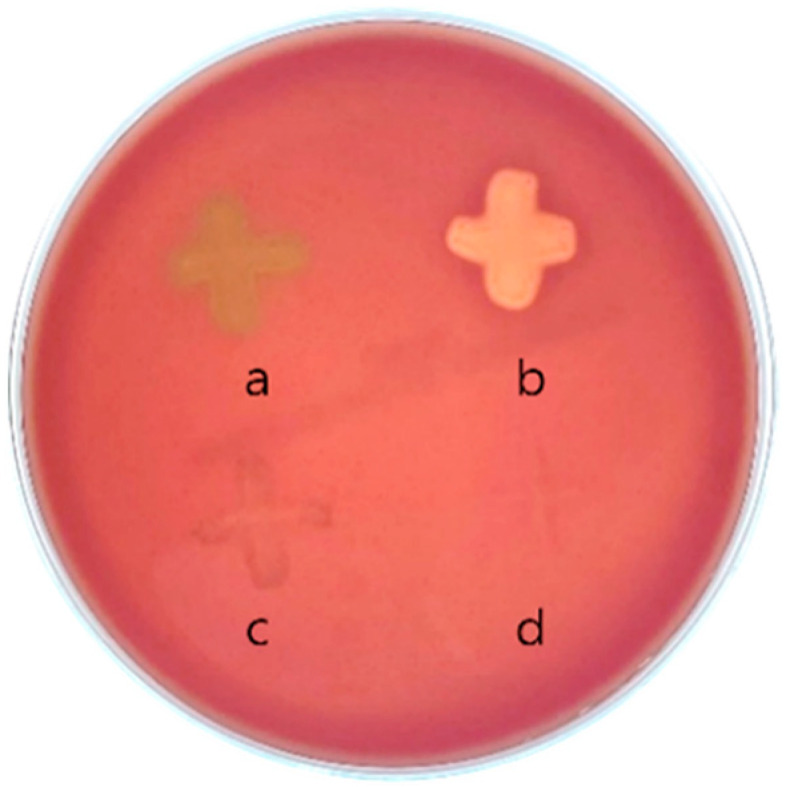
Hemolytic activity of bacterial strains. (a) *S. pneumoniae* KCCM 41570 (α-hemolysis); (b) *S. pyogenes* KCCM 40411 (β-hemolysis); (c) *S. agalactiae* KCCM 40417; and (d) *L. buchneri* KU200793 (γ-hemolysis).

*S. pneumoniae* KCCM 41570 exhibited α-hemolysis, characterized by a greenish discoloration around the colonies on blood agar (Figure 1a). In contrast, the positive control, *S. pyogenes* KCCM 40411, displayed β-hemolysis, forming a clear hemolytic zone around the colonies (Figure 1b). *S. agalactiae* KCCM 40417 showed no hemolysis on blood agar, and the medium retained its red color (Figure 1c). Similarly, *L. buchneri* KU200793 exhibited no color change around its colonies, indicating the absence of hemolytic activity, and was thus classified as γ-hemolytic (Figure 1d).

### 3.2. Bile Salt Deconjugation Activity

**Figure 2:**
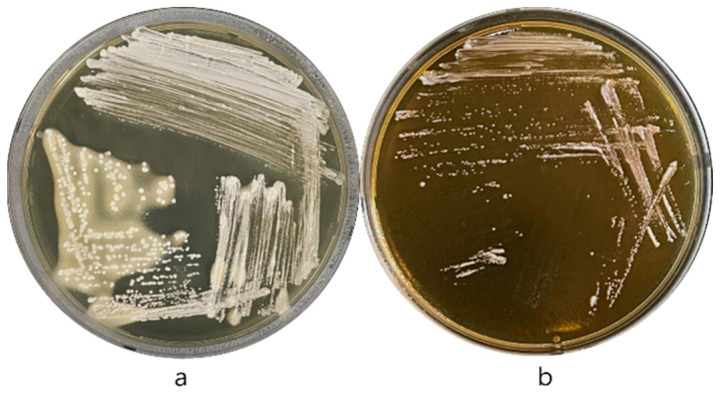
Bile salt deconjugation test. (**a**) *Lp. plantarum* KU15122 (positive control) exhibited a precipitation halo, whereas (**b**) *L. buchneri* KU200793 showed no precipitate.

*Lp. plantarum* KU15122 exhibited positive BSH activity, as indicated by the formation of a precipitation halo around the colonies due to bile salt deconjugation. Additionally, the agar appeared cloudy, and the colonies displayed a whitish, granular texture (Figure 2a). In contrast, *L. buchneri* KU200793 did not form any precipitate, confirming the absence of BSH activity (Figure 2b).

### 3.3. Cytotoxic Effects on Caco-2 Cells

**Figure 3:**
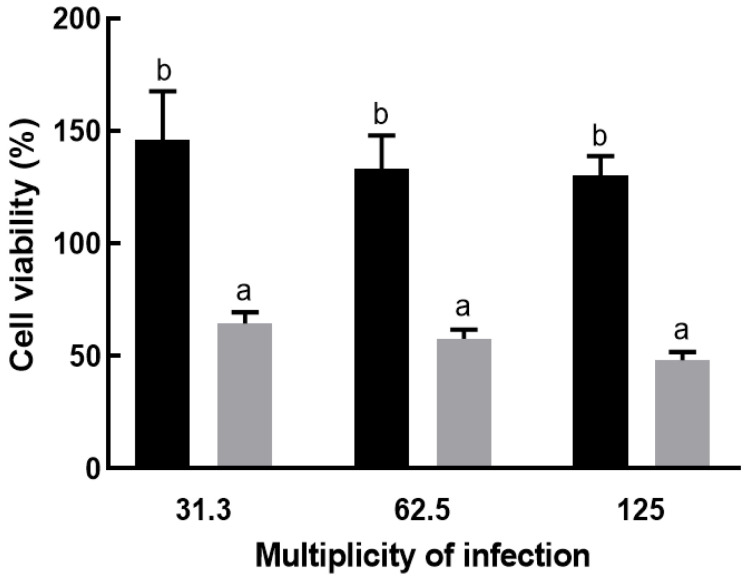
Effect of *L. buchneri* KU200793 on the cell viability of Caco-2 cells. Cells were treated with *L. buchneri* KU200793 (black bar) or *K. pneumoniae* subsp. *pneumoniae* KCCM 60022 (grey bar). Different letters indicate significant differences (*p* < 0.05).

*L. buchneri* KU200793 did not exhibit cytotoxicity under any of the tested MOI conditions. The positive control strain, *K. pneumoniae* subsp. *pneumoniae* KCCM 60022, showed cell viabilities of 64%, 58%, and 48% at MOI values of 31.3, 62.5, and 125, respectively. In contrast, *L. buchneri* KU200793 exhibited significantly higher cell viabilities of 146%, 133%, and 130%, respectively, under the same conditions, surpassing those of the untreated control group (Figure 3). These results clearly indicate that *L. buchneri* KU200793 is not cytotoxic and may even enhance cell viability under all tested MOI conditions.

### 3.4. Mucin Degradation Potential

**Figure 4:**
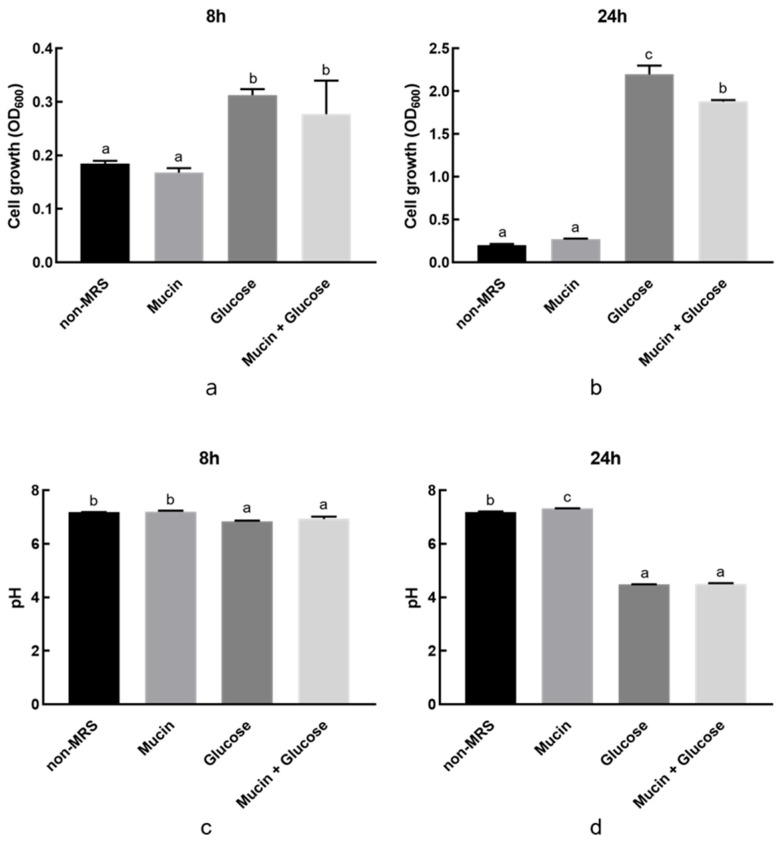
Mucin degradation activity of *L. buchneri* KU200793. (**a**,**b**) Cell growth (OD600) at 8 h and 24 h; (**c**,**d**) culture pH at 8 h and 24 h. Different letters indicate significant differences (*p* < 0.05).

*L. buchneri* KU200793 did not exhibit any increase in OD or pH changes in the medium containing 0.3% (*w*/*v*) mucin as the sole carbon source. In contrast, OD values increased in media containing glucose, which is commonly utilized as an optimal carbon source by most LAB (Figure 4). These results demonstrate that *L. buchneri* KU200793 does not exhibit mucin-degrading activity.

### 3.5. D-Lactic Acid Production Levels

The D-lactic acid production by *L. buchneri* KU200793 was quantified. The assay results showed that *L. buchneri* KU200793 produced 2 mM D-lactic acid (Table 1).

### 3.6. Biogenic Amine Production Profile

Under our experimental conditions, the analysis of BA production in *L. buchneri* KU200793 revealed the presence of spermidine at a concentration of 15.27 ppm in the supernatant. However, no detectable levels of other Bas, such as agmatine, histamine, β-phenylethylamine, putrescine, serotonin, tyramine, and tryptamine were observed (Table 2).

### 3.7. Antibiotic Resistance Pattern

**Figure 5:**
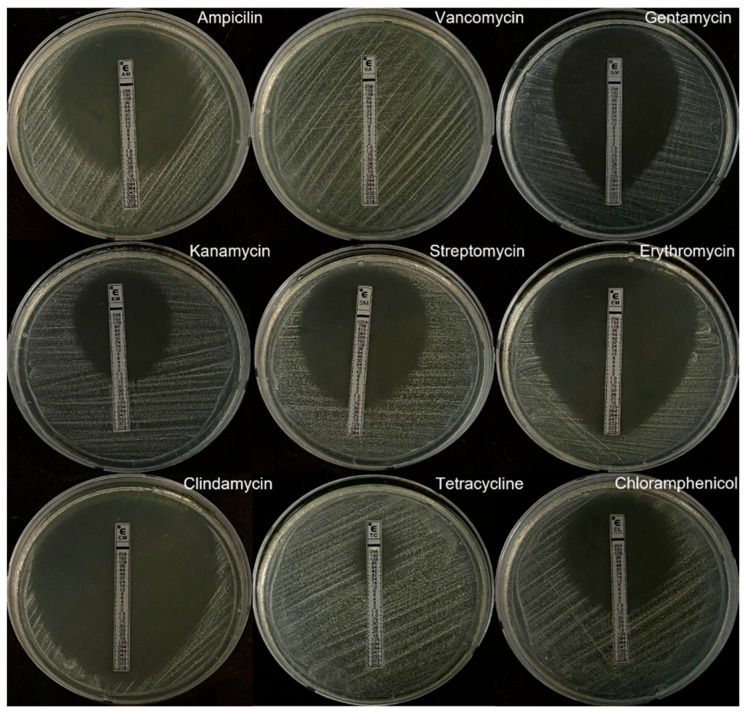
E-test for the determination of antibiotic resistance.

The antibiotic resistance of *L. buchneri* KU200793 was evaluated based on MIC values (μg/mL) exceeding the thresholds recommended by the EFSA. The results demonstrated that *L. buchneri* KU200793 was susceptible to all tested antibiotics, except for tetracycline (Figure 5, Table 3).

### 3.8. Genome-Based Identification of Resistance and Virulence Determinants

The complete genome sequence of *L. buchneri* KU200793 was analyzed to identify ARGs and assess the HGT potential. A total of 29 genes were predicted to have undergone HGT, of which 16 were identified as acquired genes and 13 as donated genes. Further analysis using HGTree2 and RGI confirmed that none of these predicted HGT-acquired genes contained ARGs. Additionally, PlasmidFinder 2.0 confirmed the absence of plasmids in *L. buchneri* KU200793. PHASTEST, which was used to evaluate the possibility of transduction, identified one intact prophage region. However, further analyses using RGI and ResFinder confirmed that no ARGs were present in the identified prophage region. The MobileElementFinder program identified eight mobile genetic elements, but ResFinder analysis did not detect any ARGs within these elements. Furthermore, RGI analysis identified *tetA*(*58*) with 33.64% homology and *nalD* with 30.17% homology (Table 4). The low-sequence homology suggested that these genes were unlikely to function as intact ARGs. Based on these results, we concluded that no functional ARGs were present within the intact prophage regions.

### 3.9. Gelatin Liquefaction Activity

No gelatin liquefaction was observed in the medium inoculated with *L. buchneri* KU200793. The medium remained solid, indicating that the strain does not produce enzymes capable of hydrolyzing gelatin. In contrast, the positive control strain, *Br. parabrevis* KCCM 41421, exhibited gelatin hydrolysis, resulting in liquefaction of the medium.

### 3.10. Urease Activity 

The urease activity of *L. buchneri* KU200793 was assessed, and no color change was observed in the medium, indicating that the strain does not produce urease. In contrast, the positive control, *P. vulgaris* KCCM 40221, induced a color shift to pink, confirming urease activity and ammonia production.

### 3.11. Indole Production 

*L. buchneri* KU200793 showed no observable changes in the surface layer after the addition of Kovac’s reagent, and no pink ring formation was detected, indicating that this strain did not produce indole. In contrast, the positive control strain, *E. coli* KCCM 11234, exhibited a distinct pink ring on the surface after the addition of Kovac’s reagent, suggesting tryptophanase activity, which enables the conversion of tryptophan to indole.

## 4. Discussion

Safety evaluation of probiotic strains is a crucial prerequisite for their commercial application and potential health benefits. Unverified strains present health risks, including opportunistic infections, HGT of antibiotic resistance, and harmful metabolic activities. Therefore, a comprehensive safety assessment—encompassing genome-based screening for ARGs, metabolic profiling, and both in vivo and in vitro toxicity evaluations—is essential. Only strains that meet rigorous safety standards should be considered for probiotic use. *L. buchneri* is listed in the EFSA’s Qualified Presumption of Safety (QPS) list, indicating its safety status for use in food and feed applications in the EU. This species-level recognition provides a basis for investigating the safety characteristics of strain *L. buchneri* KU200793.

Hemolytic activity is a key parameter in probiotic safety evaluation, as β-hemolysis is associated with pathogenic potential. Using blood agar assays, *L. buchneri* KU200793 exhibited no hemolytic activity, consistent with the γ-hemolytic phenotype. The absence of hemolytic activity indicates non-pathogenic properties in probiotic strains and serves as a positive indicator in their safety assessment [17]. Consistent with our findings, a previous study reported no β-hemolytic activity in four other *Lactobacillus* strains, including *L. fermentum* IDCC 3901 [18].

BSH is widely present in the gut microbiota and contributes to microbial survival, but its activity may generate harmful secondary bile acids linked to adverse health effects [19,20]. Owing to the potential risks associated with BSH activity, the Ministry of Food and Drug Safety of Korea recommends cautionary labeling for probiotics with positive BSH activity to prevent excessive consumption. The findings of this study confirm that *L. buchneri* KU200793 is a safe strain with no BSH activity. These results align with those of a previous study reporting the absence of BSH activity in *L. paracasei* subsp. *paracasei* NTU101 [8].

*L. buchneri* KU200793 enhanced the survival of Caco-2 cells, which are widely used as an intestinal epithelial model for probiotic safety assessment. Cell viability was assessed using a colorimetric assay with water-soluble tetrazolium-8. The amount of formazan produced correlates with the metabolic activity of viable cells, thereby indirectly reflecting cell viability. These findings provide scientific evidence supporting the safety of *L. buchneri* KU200793 and reinforce previous reports that probiotic strains are not expected to be cytotoxic to intestinal epithelial cells [21].

The intestinal mucus layer provides both a favorable niche for microbial survival and a primary barrier against pathogen and toxin entry. Mucin degradation, a known virulence trait of pathogens such as *Vibrio cholerae*, *Helicobacter pylori*, *Salmonella*, *Shigella*, and *Staphylococcus aureus*, facilitates host invasion and increases susceptibility to harmful compounds [22,23]. This study aligns with previous research reporting that *L. rhamnosus* HN001, *L. acidophilus* HN017, and *Bifidobacterium lactis* HN019 do not exhibit mucin-degradation activity [24]. Similarly, an earlier study found that none of the 75 tested *Lactococcus lactis* strains exhibited mucin-degrading activity [25].

In contrast to L-lactic acid, which is efficiently metabolized in humans, D-lactic acid is produced in a strain- and condition-dependent manner and undergoes only limited degradation by intestinal enzymes [26]. Consequently, the excessive consumption of probiotics that produce D-lactic acid can lead to its accumulation in the bloodstream, potentially resulting in D-lactic acidosis. In healthy adults, blood D-lactic acid levels typically range from 5 to 20 μmol/L, which is considered safe [27]. However, when the concentration exceeds 3 mmol/L, D-lactic acidosis may occur, causing various neurological symptoms [28]. The production levels of D-lactic acid vary depending on the type of probiotic strain, and *L. buchneri*, which belongs to the *Lentilactobacillus* genus, is known to produce both D- and L-lactic acid, classifying it as a D, L-lactate-producing strain [29]. *L. buchneri* KU200793 demonstrated a relatively low level of D-lactic acid production, highlighting its safety attributes as a probiotic strain. The amount of D-lactic acid produced by this strain was significantly lower than that produced by *L. lactis* DSM20072, which produces 7.47 mM [30], and *L. rhamnosus* GG, a widely used commercial probiotic strain [26].

BAs produced via amino acid decarboxylation are well known to act as neurotransmitters in humans, and spermidine, an endogenous metabolite, additionally plays a crucial role in cell growth, anti-aging, and autophagy [31]. Fermented and long-stored foods often show elevated BA levels due to microbial contamination, and some Lactobacillus species are major BA producers that generate them under acidic conditions as a survival strategy [32,33]. However, excessive BA accumulation may cause adverse effects such as headaches, neurological disorders, or hypotension, and high intake of histamine and tyramine has been linked to histamine poisoning and hypertensive crisis [34].

The spermidine production in *L. buchneri* KU200793 was relatively low compared with that in previously reported probiotic strains, such as *L. brevis* NZ.Lbr.082 and *L. paracasei* NZ.Lbp.105, which produced 23.06 and 35.81 ppm of spermidine, respectively [35]. Although these strains are not phylogenetically closest to *L. buchneri*, they are commonly used probiotic lactobacilli with established safety profiles and functional similarities, particularly in fermented food contexts [36]. Therefore, the comparison provides a relevant benchmark indicating that the BA production of *L. buchneri* KU200793 remains within a safe and acceptable range. Furthermore, fermented soybean-based foods contain high concentrations of BAs and BA-producing microorganisms. According to the literature, the maximum BA concentrations reported in fermented products such as soy sauce, natto, and *cheonggukjang* were 53.1 ppm, 478.1 ppm, and 79.1 ppm, respectively, indicating that the BA levels present in typical dietary intake are higher than those produced by *L. buchneri* KU200793. In addition, no specific dietary intake recommendations or toxicity thresholds have been established for putrescine, spermidine, or tryptamine in healthy individuals, suggesting that their consumption is not strictly regulated. Moreover, the regulatory authorities in various countries have not set specific permissible limits for spermidine, further supporting its safety [37].

In nature, antibiotics function as competitive tools among microorganisms, but their indiscriminate use has accelerated the emergence of antimicrobial resistance in both pathogenic and commensal species [38]. LAB, including *Lactobacillus* species, are generally considered safe. However, concerns regarding antibiotic resistance in LAB have emerged following reports of antibiotic-resistant *Lactobacillus* strains isolated from cheese [39]. The acquisition of antibiotic resistance reduces treatment efficacy, and the potential transfer of ARGs from probiotics to pathogens represents a critical public health concern. Therefore, assessing the antibiotic resistance profiles of probiotic strains before they can be used for food applications is essential [40]. In the present study, *L. buchneri* KU200793 exhibited tetracycline resistance; however, its overall antibiotic resistance levels were lower than those previously reported for other probiotic strains. *L. salivarius* CGMCC2070 showed resistance to both tetracycline and erythromycin as well as intermediate resistance to gentamicin [41]. In addition, five *L. lactis* strains demonstrated resistance to both tetracycline and streptomycin [42]. Compared with previously reported strains that exhibit resistance to multiple antibiotics, *L. buchneri* KU200793 appears to be a relatively safer probiotic candidate regarding antibiotic resistance. However, further investigations are required to clarify the mechanisms and genomic context of tetracycline resistance, for which we conducted in silico analysis based on whole-genome sequencing data.

Antibiotic resistance can arise either intrinsically, through mutations stably inherited within a strain, or via HGT, which poses a greater risk due to the possibility of ARG dissemination to pathogens [43,44]. Plasmid conjugation, whereby a donor strain transfers a plasmid to a recipient through pilus-mediated contact, represents a major HGT mechanism [45]. Transduction by bacteriophages and mobilization of transposons further contribute to ARG transfer, and integration of resistance-associated transposons into plasmids can accelerate ARG dissemination among bacterial populations [46,47]. Genome analysis confirmed the absence of exogenous resistance determinants commonly associated with HGT, indicating that the tetracycline resistance in *L. buchneri* KU200793 is intrinsic rather than acquired. This result suggests that the strain has a minimal potential for ARG dissemination via horizontal gene transfer.

Gelatinase is a biologically active protease that degrades host proteins such as collagen, casein, and hemoglobin, and its activity has been implicated in bacterial pathogenesis, tissue damage, and the progression of inflammatory diseases such as endocarditis [24,48]. In addition, gelatinase can modulate host factors including cytokines and growth factors, functioning as a virulence determinant in pathogenic bacteria, while bacterial metalloproteinases have also been linked to infection processes and tumor progression [49]. In this study, *L. buchneri* KU200793 showed no gelatin liquefaction, indicating the absence of gelatinase activity and supporting its safety by excluding this virulence-associated trait.

Urease is a nickel-dependent metalloenzyme that hydrolyzes urea into carbon dioxide and ammonia, and its activity has been implicated in urinary tract infections, hepatic encephalopathy, and other pathological conditions [50,51]. Moreover, in *H. pylori*, urease contributes to acid resistance but also causes cytotoxic damage to gastric epithelial cells through ammonia production. In this study, *L. buchneri* KU200793 showed no detectable urease activity, supporting its safety by excluding this virulence-associated trait.

Indole is a nitrogen-containing metabolite generated by microbial tryptophan metabolism via tryptophanase and has been associated with neurotoxic and metabolic effects, including impaired motor function, anxiety, and depression [52]. Once absorbed, indole is converted to indoxyl sulfate in the liver, which induces nephrotoxicity and has been reported at elevated levels in patients with Parkinson’s disease [52,53]. In this study, *L. buchneri* KU200793 did not produce indole, consistent with previous findings that none of the tested *Lactobacillus* strains exhibited this activity [54].

## 5. Conclusions

A comprehensive safety assessment of *L. buchneri* KU200793 demonstrated its potential as a safe probiotic strain. The absence of harmful enzymatic activities, the low production of detrimental metabolites, and the lack of cytotoxicity in Caco-2 cells collectively support its safety. Although tetracycline resistance was detected, genome analysis indicated that this trait is intrinsic and unlikely to be transferred horizontally, minimizing concerns about ARG dissemination. In addition to the previously reported neuroprotective properties of this strain, the present study established its safety, thereby reinforcing the feasibility of its use in food applications and probiotic supplementation. Overall, these findings highlight the importance of rigorous safety evaluations to ensure the responsible development of probiotics and support the potential utilization of *L. buchneri* KU200793 as a safe candidate for incorporation into dietary products.

## Figures and Tables

**Table 1:** D-Lactic acid production of *Lentilactobacillus buchneri* KU200793.

Strain	D-Lactic Acid (mM)
*L. buchneri* KU200793	2.0 ± 0.3

The data are expressed as mean ± SD (*n* = 3).

**Table 2:** Biogenic amine production of *Lentilactobacillus buchneri* KU200793.

Biogenic Amines	Concentration (ppm)
Agmatine	n.d. *
β-phenylethylamine	n.d.
Putrescine	n.d.
Histamine	n.d.
Serotonin	n.d.
Spermidine	15.3 ± 7.0
Tryptamine	n.d.
Tyramine	n.d.

* n.d.: Not detected. The data are expressed as mean ± SD (*n* = 3).

**Table 3:** Minimum inhibitory concentration and antibiotic susceptibility of *Lentilactobacillus buchneri* KU200793.

Antibiotics	Cut-Off Value (µg/mL) *	MIC (µg/mL)	Assessment
Ampicillin	2	0.38	S ***
Vancomycin	n.r. **	-	-
Gentamycin	16	0.047	S
Kanamycin	32	1	S
Streptomycin	64	0.5	S
Erythromycin	1	0.016	S
Clindamycin	1	0.016	S
Tetracycline	8	48	R ****
Chloramphenicol	4	1	S

* Cut-off value based on European Food Safety Authority guidelines; ** n.r., not required; *** S, Susceptible; **** R, Resistant.

**Table 4:** Results of antibiotic resistance gene analysis based on genome sequence data.

RGI Criteria	ARO Term	AMR Gene Family	Drug Class	% Identity Matching Region
Loose	*tetA*(*58*)	Major facilitator superfamily antibiotic efflux pump	tetracycline antibiotic	33.64
	*nalD*	Resistance-nodulation-cell division antibiotic efflux pump	macrolide antibiotic, fluoroquinolone antibiotic, monobactam, carbapenem, cephalosporin, cephamycin, penam, tetracycline antibiotic, peptide antibiotic, aminocoumarin antibiotic, diaminopyrimidine antibiotic, sulfonamide antibiotic, phenicol antibiotic, penem	30.17

## Data Availability

The original contributions presented in this study are included in the article. Further inquiries can be directed to the corresponding authors.
